# Uric Acid as a Marker of Kidney Disease: Review of the Current Literature

**DOI:** 10.1155/2015/382918

**Published:** 2015-05-27

**Authors:** Christin Giordano, Olga Karasik, Kelli King-Morris, Abdo Asmar

**Affiliations:** ^1^Department of Internal Medicine, University of Central Florida, College of Medicine, Orlando, FL 32827, USA; ^2^Orlando VA Medical Center, Orlando, FL 32827, USA

## Abstract

Uric acid has been implicated in the pathophysiology of renal disease; however renal clearance makes a causal relationship difficult to prove. We examine the current literature to support a potential role of uric acid in the development of kidney disease and to determine the potential to use uric acid as a marker for future renal decline. After review, we conclude that uric acid is definitively linked to the development of chronic kidney disease and can be a poor prognostic factor for the development of acute renal failure, as well. However, further human research is needed before predictive models utilizing uric acid can be developed and used in the clinical setting.

## 1. Introduction

Uric acid is the final oxidation product of purine metabolism and is renally excreted [1]. Therefore, elevated serum uric acid levels are seen in patients with reduced glomerular filtration rate (GFR). However, in recent years, it has been proposed that uric acid itself plays a causal role in the pathophysiology of chronic kidney disease and possibly in acute kidney injury. Review of the literature demonstrates uric acid-related cellular changes that contribute to renal disease. Thus far, it remains unclear whether these changes are reversible upon treatment of hyperuricemia. It also remains unclear whether uric acid levels can be a marker of impending renal decline.

## 2. Pathophysiology of Uric Acid in Development and Progression of Renal Disease

Studies performed on rats have demonstrated that, in the presence of hyperuricemia, there are fundamental changes in the renal vasculature. Ryu et al. found that uric acid decreased the expression of E-cadherin in epithelial cells resulting in a loss of cell-to-cell contact in the renal tubular cells of rats. Without cell-to-cell contact, epithelial cells are unable to coordinate efforts to secrete substances needed to increase renal blood flow such as nitric oxide [2]. In addition, a recent study utilizing immortalized proximal tubular epithelial cells from normal adult human male kidney has demonstrated that increasing levels of uric acid causes NAPDH-dependent oxidative changes which promote apoptosis [3]. This finding sheds light on the connection between hyperuricemia and tubulointerstitial renal damage. Further, Sánchez-Lozada et al. established that rats with increased serum uric acid levels had renal biopsies demonstrating afferent arteriolar thickening. Thickening of these arterioles decreases renal blood flow [4]. This endothelial dysfunction can be indirectly assessed by ultrasonographic flow-mediated dilatation [5].

Kanbay et al. discovered that, in over 250 patients with CKD stages 3–5, those with higher serum uric acid levels had higher systolic blood pressures, C-reactive protein levels, lower eGFR, and lower flow-mediated dilatation. Multiple logistic regression analyses confirmed an independent inverse relationship between serum uric acid levels and flow-mediated dilatation confirming that endothelial function was directly affected by serum uric acid levels in patients with nondiabetic nephropathy (*p* ≤ 0.001) [5]. Similarly, a later study by Turak et al. examined 112 patients with essential hypertension. Patients without baseline renal dysfunction had statistically higher baseline serum uric acid levels compared to those in the control group, suggesting a causal relationship between serum uric acid level and the development of essential hypertension, regardless of baseline renal function and therefore unrelated to renal clearance [6]. When taken together, these four studies demonstrate that, in both animal and human models, uric acid levels are inversely related to endothelial function, resulting in afferent arteriolar thickening and a decrease in vasodilatation which are known to be part of the pathophysiology of worsening renal function.

Moreover, hyperuricemia, along with a decrease in the number of nephrons, has been implicated in the impaired autoregulation seen in hypertension. Studies have demonstrated that chronic hyperuricemia leads to salt-sensitivity, which may be a response to the reduced renal blood flow seen in hypertension. Thus, the exact relationship between hyperuricemia and hypertension is difficult to establish; it is unclear whether hyperuricemia leads to hypertension, via increased sodium avidity, or whether one merely potentiates the other [7]. Endothelial function was shown to improve with the use of xanthine oxidase inhibitors to reduce serum uric acid levels, but this was not the case with the use of other agents such as probenecid, which instead increases uric acid urinary excretion [8]. Allopurinol was found to result in lower serum uric acid levels, as well as improve renal function [9]. Therefore, it appears plausible that xanthine and xanthine oxidants may contribute to vascular dysfunction in addition to, or in place of, uric acid in states of hyperuricemia and hypertension.

Another proposed mechanism for uric acid to elicit renal damage is via fructose. Fructokinase is expressed primarily in the proximal renal tubule and in the liver [10]. Uric acid increases fructose's ability to increase fat stores, which is thought to be the underlying mechanism of the association between elevated uric acid levels, metabolic syndrome, and fatty liver disease [1]. Cirillo et al. found that fructose, when metabolized by fructokinase, generates both oxidants and uric acid, which induces proximal tubular injury [11]. Specifically, fructose simulates chemokine monocyte chemotactic protein-1 in proximal tubular cells, which increases macrophage and monocyte presence leading to damage [12, 13]. A study performed with fructokinase knockout mice demonstrated protection from developing diabetic nephropathy, suggesting that this process may be mediated by the endogenous production of fructose. The knockout mice had less cortical uric acid accumulation than the wild-type mice [10]. Thus, mice with lower cortical uric acid were protected from developing nephropathy.

Uric acid is known to cause endothelial dysfunction, vascular smooth muscle cell proliferation, increased IL-6 synthesis, and impairment of nitric oxide production, all of which may contribute to the progression of chronic kidney disease [9]. In fact, Johnson et al. observed that uric acid levels were elevated in hypertensive populations who had increased risk of progression of kidney disease including African Americans, patients with gout, patients with chronic lead ingestion, those with metabolic syndrome, and those with chronic diuretic use [14]. Thus, uric acid has been shown in both animal and human models to adversely affect endothelial function, increase the risk for hypertension, and possibly increase the risk for nephropathy.

## 3. Hyperuricemia and Risk of Development of Renal Disease

As demonstrated above, hyperuricemia has been shown to cause changes in renal physiology. We must then investigate whether these changes translate to increased risk for renal disease. Chonchol et al. utilized a prospective cohort study, the Cardiovascular Health Study, which included over 4600 subjects who had a serum uric acid level and GFR analyzed. The main cohort had uric acid level and GFR measured at baseline and at years 2, 5, and 9, whereas the African American cohort had these levels measured at study years 5 and 9 only. Decrease in renal function was defined as an annual decrease in GFR of at least 3 mL/min/1.73 m^2^ and chronic kidney disease was defined as an estimated GFR (eGFR) less than 60 mL/min/1.73 m^2^ at year 5 for the main cohort and year 9 for the African-American cohort. The average age of the cohort was 73 while the average eGFR was 78 mL/min/1.73 m^2^ and uric acid level 5.7 mg/dL (serum uric acid reference range: 3.4–7.2 mg/dL). Participants were then divided into 5 groups by uric acid level: ≤4.40 mg/dL, 4.41–5.20, 5.21–5.90, 5.91–6.90, and ≥6.91. The odds of developing an eGFR of less than 60 mL/min/1.73 m^2^ over the study period were linearly associated with increasing levels of uric acid with odds ratio of 1.0, 1.71 (95% CI, 1.37 to 2.64), 2.06 (95% CI, 1.60 to 2.64), 2.99 (95% CI, 2.34 to 3.83), and 6.72 (95% CI, 5.13 to 8.78), respectively, on a cross-sectional basis. However, uric acid level at baseline was not associated with the development of chronic kidney disease [15].

On the other hand, Iseki et al. analyzed data from over 6,000 Japanese subjects who participated in a health screening twice, 2 years apart. High serum creatinine was defined as a serum creatinine ≥1.4 mg/dL in men and ≥1.2 mg/dL in women. Serum uric acid levels ≥5 mg/dL at initial screening of subjects with normal serum creatinine had a relative risk of 1.351 for developing high serum creatinine. However, serum uric acid levels ≥8 mg/dL with normal renal function at initial screening had a relative risk of 2.91 in men and 10.39 in women for developing a high serum creatinine two years later. The authors concluded that serum uric acid levels might be reasonable to determine which patients were at higher risk for developing worsening renal function in the near future [16].

Finally, Weiner et al. performed a prospective cohort study following over 13,000 people with normal kidney function (mean eGFR = 90.4 mL/min/1.73 m^2^) and found that 7.9% of the cohort developed renal disease by follow-up at 8.5 years. Logistic regression models determined that a baseline elevated serum uric acid level predicted worsening renal function irrespective of age, gender, race, diabetes, hypertension, alcohol use, smoking, lipids, and baseline renal function [17].

## 4. Hyperuricemia and Progression of Disease and Mortality

While hyperuricemia may or may not predispose a patient to developing de novo renal disease, studies have indicated that the development of hyperuricemia leads to progression of existing renal disease and an increase in mortality. Odden et al. divided 10,956 patients into three groups based on sex-specific lowest, middle, and highest percentiles of uric acid levels (<25th, 25th–75th, and >75th) with a final outcome of cardiovascular death and all-cause mortality. The lowest risk of cardiovascular and all-cause mortality occurred in women with the lowest uric acid levels, while the highest risk for cardiovascular and all-cause mortality occurred in both men and women with the highest uric acid levels. However, once renal function was accounted for, this no longer held true and there was no statistical difference for the risk for cardiovascular and all-cause mortality between those with high, intermediate, and low uric acid levels. This may suggest that eGFR and uric acid are inherently linked and may be in the same causal pathway affecting cardiovascular mortality [18].

Weiner et al. examined approximately 1600 participants who had an eGFR between 15 mL/min/1.73 m^2^ and 60 mL/min/1.73 m^2^ and had a mean follow-up of approximately 9 years with approximately half of participants reaching one of the primary endpoints of myocardial infarction (MI), stroke, and all-cause mortality. While C-reactive protein increases had a statistically significant hazard ratio for all-cause mortality, serum uric acid increases had a trend towards increasing all-cause mortality, without reaching statistical significance. Patients with an increase in their uric acid levels may have an increased all-cause mortality. However, eGFR was not repeated at follow-up and thus conclusions cannot be made about whether increases in serum uric acid predicted worsening disease [19].

Syrjänen et al. followed up 223 patients diagnosed with IgA nephropathy from time of renal biopsy for a median of 10 years, with 18% of the patients demonstrating progression of disease with either an increase in serum creatinine over 20% over baseline or 125 micromol/L in men or 105 micromol/L in women. In patients with progressive disease, proteinuria, hypertension, hypertriglyceridemia, and hyperuricemia were more common at the time of renal biopsy than in those patients who did not progress. This effect was present even if the patient had normal renal function at time of biopsy. The relative risk for all patients with hyperuricemia at baseline for progressive disease and for patients who had initially normal renal function was 2.2 and 2.7, respectively. Further, and perhaps more alarmingly, survival rate curves for those with nonprogressive disease demonstrated that baseline hyperuricemia predicted worse overall survival [20].

This effect extended to those with severe renal dysfunction requiring dialysis. Suliman et al. studied patients who were starting renal replacement therapy with a primary endpoint of mortality. The researchers divided the patients into quintiles based on serum uric acid levels. They found that the highest hazard ratios for mortality existed in the group with the highest serum uric acid level (>8.9 mg/dL), with a hazard ratio of 1.96 (95% CI, 1.10 to 3.48; *p* = 0.02) [21].

Assuming that hyperuricemia leads to progression of disease and worsening mortality, can treatment of the increased uric acid level change the natural course of disease?

Utilizing an in vivo protocol with 54 rats, Ryu et al. found that the rats who had induced hyperuricemia developed renal interstitial fibrosis but that rats with hyperuricemia who were subsequently treated with allopurinol did not have an increase in tubulointerstitial fibrosis over the course of several weeks. The rats were not followed up for effects on overall mortality [2].

Goicoechea et al. performed a prospective randomized trial with over 100 patients with an eGFR <60 mL/min and found that patients treated with allopurinol had significantly reduced serum uric acid and C-reactive protein levels. Further, the eGFR did not have a significant change in the allopurinol-treated patients (from 40.8 to 42.2 mL/min/1.73 m^2^) but worsened in nontreated patients (from 39.5 to 35.9 mL/min/1.73 m^2^) over a 24-month period. While this reached a statistically significant difference between the two groups (*p* = 0.000), it is unclear if this was of clinical significance as the decrease in eGFR in the latter group was marginal. However, patients in the allopurinol group had fewer cardiac events on a Kaplan-Meier survival curve over a mean follow-up time of 23.4 months (log rank: 4.25; *p* = 0.039). While lowering serum uric acid levels may not have a clinical significance for renal function, it may provide a cardiac survival benefit [22].

While uric acid levels have been associated with the progression of renal disease in patients with chronic renal disease, the same was not found in those who had received a renal transplant. Meier-Kriesche et al. studied 1645 postrenal transplant patients in a prospective cohort study and divided patients into 3 groups based on their serum uric acid levels: ≥6.4 mg/dL, 4.4–6.3 mg/dL, and ≤4.3 mg/dL. They then analyzed differences in renal function three years after renal transplant and found that when renal function at 1 month after transplant was accounted for, there was no statistical association between uric acid level and renal function three years after transplant (*p* = 0.62) [23]. There was no study found in the literature that addressed the effect on overall mortality in the posttransplant patient. Further research in this area is needed to help determine, while renal function was no different several years from transplant, if there is potentially a mortality benefit to lowering uric acid levels in the posttransplant setting. This is especially true given the high cardiac-associated mortality rates in the posttransplant setting, previously attributed to immunosuppression and more rapid progression of atherosclerosis [24]. In addition, immunosuppressive agents themselves, such as cyclosporine, can contribute to hyperuricemia in this setting, with one study demonstrating an incidence of 80% of patients on cyclosporine and prednisone and 55% of patients treated with azathioprine, prednisone, and antilymphocytic globulin (*p* ≤ 0.002) [25].

The Losartan Intervention for Endpoint reduction in hypertension (LIFE) study is a large epidemiologic study of over 9000 patients with hypertension and electrocardiographic changes consistent with left ventricular hypertrophy with a mean follow-up of 4.8 years. Høieggen et al. used data obtained to analyze the effects of serum uric acid on cardiovascular outcomes including cardiovascular death, fatal or nonfatal myocardial infarction, and fatal or nonfatal stroke. Subanalysis of the data found that women, but not men, had a statistically significant association between baseline serum uric acid levels and cardiovascular outcomes with a hazard ratio of 1.025 (1.013–1.037), *p* < 0.0001. However, such a small hazard ratio may not be clinically significant. The study then compared outcomes of patients who received a beta-blocker, atenolol, and those who received an angiotensin II receptor antagonist which also decreases serum uric acid, losartan. Losartan, when compared to atenolol, not unsurprisingly, attenuated the increase of serum uric acid over several years and resulted in lower cardiovascular mortality rates. As the authors pointed out, the LIFE study was not designed to measure this particular outcome and thus the results should be repeated in a dedicated randomized control study [26].

A small study dedicated to the study of allopurinol effects on left ventricular mass in patients with CKD enrolled 67 patients and compared patients who received allopurinol versus placebo after 9 months of therapy. At baseline, both groups had similar left ventricular mass, estimated GFR, and serum uric acid level. There was a statistically significant decrease of 5% in left ventricular mass index in patients receiving allopurinol (*p* = 0.036) and improvement in flow-mediated dilation (*p* = 0.009). Of interest, those in the allopurinol group were more likely to be taken off antihypertensives as their blood pressure normalized. However, despite these effects, no correlation between urate levels and changes seen in left ventricular mass and flow-mediation dilation was seen. This calls into question the role of uric acid in the development of left ventricular hypertrophy and even in endothelial dysfunction which was previously shown to be inversely associated with uric acid levels [27]. In fact, Butler et al. performed a study to address the effects of allopurinol on endothelial dysfunction utilizing bilateral venous occlusion plethysmography specifically in patients with type 2 diabetes mellitus with stage 1 hypertension. The study team compared patients to age-matched healthy controls and found that, after a period of 1 month on allopurinol, patients experienced a near-normalization of endothelial function when compared to placebo. The main limitations of this study include the limited length of time and the small study size of only 11 patients with diabetes and 12 healthy participants [28].

## 5. Uric Acid Levels and Acute Kidney Injury

Uric acid association with acute kidney injury was first demonstrated in tumor lysis syndrome. However, it is now known that even when uric acid levels are not high enough to induce intrarenal crystal deposition, it may still result in acute kidney injury [29]. Lapsia et al. performed a retrospective study on 190 postoperative patients comparing the incidence of acute kidney injury at different levels of serum uric acid. They found that serum uric acid levels ≥5.5 mg/dL, ≥6 mg/dL, and ≥7 mg/dL were associated with odds ratios of developing acute kidney injury of 4.4 (95% CI, 2.4 to 8.2), 5.9 (95% CI, 3.2 to 11.3), and 39.1 (95% CI, 11.6–131.8), respectively. However, very low uric acid levels (<2.5 mg/dL) were also associated with increased odds of the development of acute kidney injury, demonstrating a J-shaped curve for AKI incidence for hypo- and hyperuricemia. Further, serum uric acid levels ≥7 mg/dL were associated with statistically significant longer hospital stays (32 days versus 18.5 days, *p* = 0.058) as well as longer duration of mechanical ventilation support (20.4 days versus 2.4 days, *p* = 0.001) [30].

Similarly, Ejaz et al. performed a prospective observational study of 100 consecutive patients after cardiac surgery in order to assess for an association serum uric acid with acute kidney injury (AKI). Serum uric acid levels were measured 24 hours after surgery. Overall, 27% of patients developed AKI with no difference in preoperative eGFR. There was no statistical difference in mean decrease in mean arterial pressure between the group that developed AKI and the group that did not. However, serum uric acid levels 24 hours postoperatively did vary statistically significantly with reported rates of 6.4 ± 0.3 mg/dL and 4.9 ± 0.1 mg/dL, respectively (*p* < 0.001). Further, the researchers divided the patients into three groups based on serum uric acid levels of ≤4.53 mg/dL, 4.54–5.77 mg/dL, and ≥5.78. They found that the incidence of AKI increased from the lowest to highest tertile of serum uric acid level: 15.1%, 11.7%, and 54.5%, respectively (*p* ≤ 0.001) [31].

Finally, Ejaz et al. performed a double blind, placebo-controlled randomized trial to assess whether preoperative treatment of hyperuricemia with rasburicase would result in a decreased incidence of acute kidney injury. Indeed, treatment with rasburicase resulted in an overall trend toward a decrease in incidence of acute kidney injury (7.7% versus 30.8%). However, because this was a pilot study, the *p* value was not statistically significant in the overall population. Despite this, in a subset of patients with an eGFR of 45 mL/min/1.73 m^2^ or less, treatment with rasburicase resulted in a statistically significant decrease in postoperative acute kidney injury incidence (0% versus 75%, *p* = 0.033) [32].

## 6. Conclusion

Multiple studies have demonstrated that uric acid is a potential causative agent of worsening renal function. Elevations in uric acid levels have been shown to change the fundamental architecture of renal histology and thus have been implicated in both the acute and chronic renal failure. While uric acid level has sufficiently been shown to have a direct correlation with progressive renal disease, can it be used reasonably as a marker of disease?

Disease markers can miss the mark for four possible reasons. The marker may not be in the causal pathway of the disease, there may be multiple causal pathways of disease of which the proposed marker accounts for only a small proportion of the pathophysiology of the disease, the marker may be unaffected by the proposed clinical intervention even though the intervention improves disease, or the clinical intervention may have effects independent of the disease, which may or may not change the marker [33]. Over the course of this review, we have demonstrated that uric acid does indeed affect endothelial function and can contribute to worsening renal disease. In addition, at least one study has shown that uric acid may be a surrogate marker for eGFR in terms of cardiovascular mortality. Some studies stated also found that reducing uric acid levels reduced the progression of renal disease. However, despite the work done thus far in hyperuricemia and its effects on hypertension and potential effects on mortality, the 2012 Kidney Disease Improving Global Outcomes practice guidelines for the evaluation and management of chronic kidney disease state that there is insufficient evidence to recommend the use of medications such as allopurinol to delay the progression of CKD [34].

Overall the challenge remains that the significance of elevations in uric acid is difficult to assess in those with chronic kidney disease because, as clearance decreases, levels of serum uric acid naturally increase. While evidence for treating asymptomatic hyperuricemia may be lacking, hyperuricemia may be used as a disease marker for the potential to develop renal disease in the future as well as predict risk for a patient with renal disease to develop worsening renal function.
